# Mind the mind: How to effectively communicate about cognition in social–ecological systems research

**DOI:** 10.1007/s13280-018-1099-7

**Published:** 2018-09-22

**Authors:** Anna Lena Bercht, Nanda Wijermans

**Affiliations:** Stockholm Resilience Centre, Stockholm University, Kräftriket 2B, 10691 Stockholm, Sweden

**Keywords:** Behaviour, Cognition, Miscommunication, Rationality, Social–ecological systems research

## Abstract

Social–ecological systems (SES) research underlines the tremendous impact of human behaviour on planet Earth. To enable a sustainable course of humanity, the integration of human cognition in SES research is crucial for better understanding the processes leading to and involved in human behaviour. However, this integration is proving a challenge, not only in terms of diverging ontological and epistemological perspectives, but also—and this has received little attention in SES research—in terms of (lacking) precision of communication regarding cognition. SES scholars often implicitly disagree on the meaning of this broad concept due to unexpressed underlying assumptions and perspectives. This paper raises awareness for the need to communicate clearly and mindfully about human cognition by exemplifying common communication pitfalls and ways of preventing them. We focus on the concept of cognition itself and provide aspects of cognition that need to be communicated explicitly, i.e. different objects of investigation and levels of description. Lastly, we illustrate means of overcoming communication pitfalls by the example of rationality.

**Electronic supplementary material:**

The online version of this article (10.1007/s13280-018-1099-7) contains supplementary material, which is available to authorised users.

## Introduction

The need to integrate human cognition in social–ecological systems (SES) research is increasingly debated at scholarly conferences, workshops and meetings. In such settings, motivated scientists and practitioners from diverse backgrounds come together to exchange relevant ideas. They often employ the term “cognition” or cognition-related concepts such as perception and rationality. The obvious ontological and epistemological challenges for such dialogues have been highlighted in relation to the various definitions of and approaches to cognition across disciplines (e.g. Gershenson 2003; von Kenemans and Ramsey 2013). As an advancement of this discussion, we stress that *the way* we *communicate* about cognition is much more complex than it may seem. While “cognition” seems intuitive and familiar, the concept not only means different things to different people, but it actually can be a wide variety of things, making it difficult to grasp. Scholars and practitioners are thus likely to misunderstand each other, often without noticing it at first. As a consequence, the vulnerable process of working towards effective inter- and transdisciplinary groups is harmed by wasting time on talking past one another and by being confused, thus draining momentum. It further leads those interacting to potentially disregard (other) explanations that may be essential for pushing science frontiers together.

We argue that this failure in communication results not only from the complexity and ambiguity of the concept(s) of cognition, but also—and especially—from what is left unsaid in the communication about cognition, exacerbating the challenges for those teaming up to tackle SES problems.

### Putting miscommunication into context

**Fig. 1:**
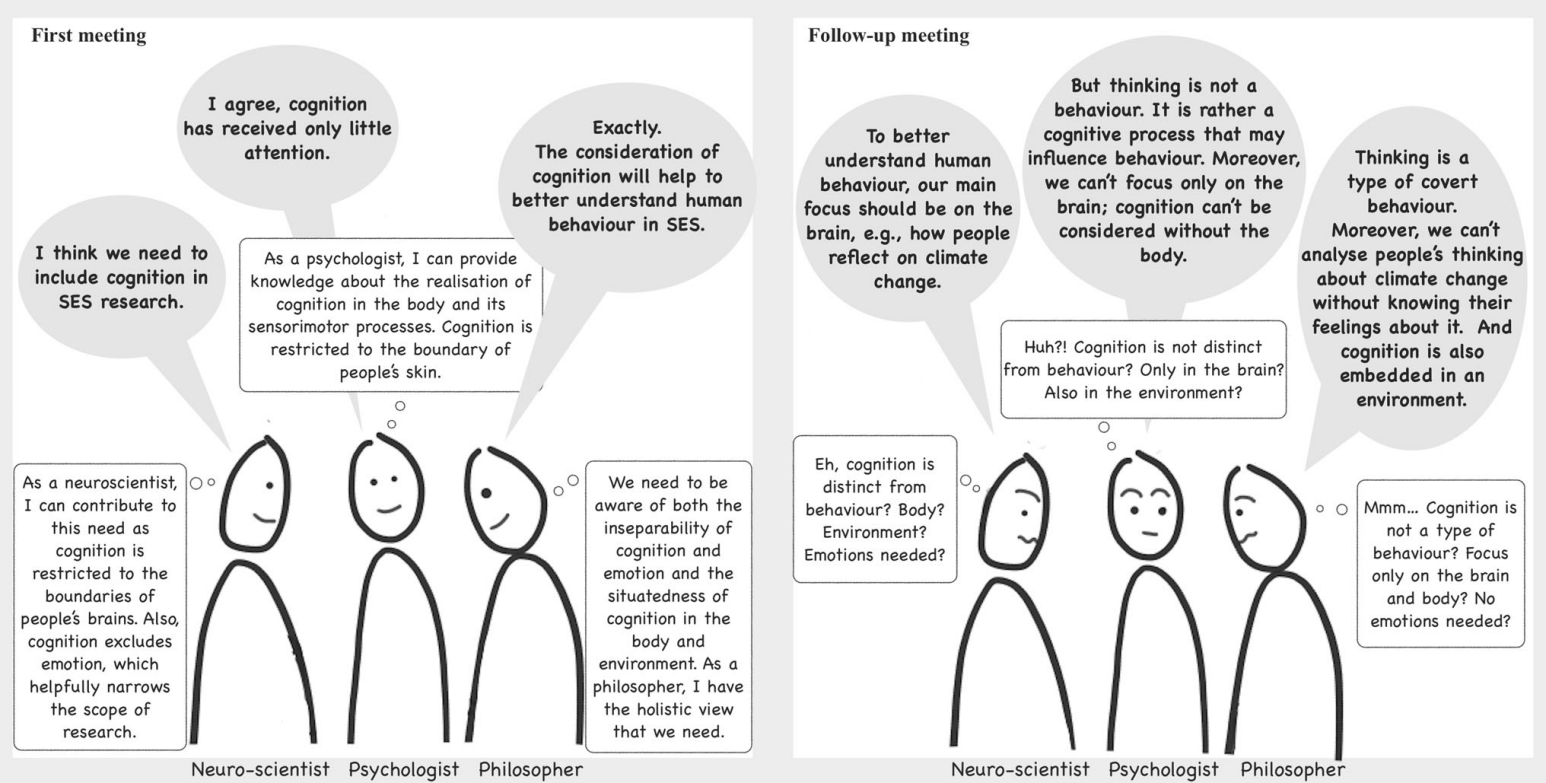
Three SES researchers misunderstanding each other without realising it at first. *Note* For demonstration purposes only, we illustrate scientists from different disciplines. However, we acknowledge that miscommunication about cognition also occurs within disciplines. *Source* Own drawing

The manifold communication pitfalls in using the concept of cognition (e.g. falsely assuming others have the same understanding of cognition as oneself, Lande and Wanlass 2015 on rehabilitation professionals; not clarifying what one means by cognition, ibid; viewing cognition as a unitary construct, Guilmette et al. 2008 on neuropsychologists) have neither been adequately addressed in the cognition literature nor have they been recognised in cognition-related SES research. Given this lack of attention and absence of examples in the literature, we use a fictitious and simplified, yet symbolic and representative dialogue between three researchers (example 1, see Fig. 1 and text below), and a real-life example from own and SES colleagues’ research experiences (example 2, see text in Box 1) to contextualise our discussion and to illustrate the impact of poor communication on conducting collaborative research, which often results in conflicting interpretations and misunderstandings and confusion.

**Table:** 

**Box 1** Real-life example from own and SES colleagues’ research experiences to illustrate the impact of poor communication on conducting collaborative research	
In a project about mental barriers to climate change action (author 1), we struggled for several weeks to develop a shared analytical framework. We literally wasted time talking past each other. This was mainly because we did neither specify our different views on the characteristics of mental barriers, nor did we clearly explain the related reasoning for our approach preferences (descriptive or normative). We superficially agreed that mental barriers refer to cognitive, emotional and/or motivational processes in the human mind that interfere with human perception and appraisal, and that they keep people from performing a specific action or changing their behaviour. *Tacit conflicting interpretations* Misunderstandings and confusion arose, however, when the advocates of a descriptive approach aimed to neutrally describe how mental barriers hinder, stop, delay or divert climate action. These researchers implicitly did not consider barriers as inherently “good” or “bad”: instead assuming the unfolding of the barrier’s effect to be context-dependent. The advocates of a normative approach, however, looked at the issue from a normative standard or rationality point of view and aimed to study the systematic patterns in terms of deviation from that standard. They implicitly considered barriers as biases, i.e. a priori maladaptive missteps in a mental process. Accordingly, the “descriptivists” assumed, for example, that (short-term interpretive) denial of climate change can be beneficial as an initial response to, for example, fear-inducing flood damage. Denial enables the mind to (unconsciously) control panic and absorb shocking or distressing information at a pace that will not plunge the person into psychological disequilibrium (see Bercht 2018 for more detail). In contrast, the “normativists” regarded denial a priori as irrational since it prevents a person from trying more productive coping activities (provided a situation can be improved). These discrepancies hampered the framing of our research questions (e.g. including or excluding questions about the potential benefits of mental barriers) and led to misinterpretations (e.g. confusing people’s denial of climate change with the belief that climate change is not real). *Problem solving* We finally resolved our unexpressed differences (e.g. denial is a priori maladaptive vs. denial can be both beneficial and maladaptive) by using a surprisingly simple technique. By jointly listing and specifying all key research elements on a whiteboard such as (a) approach (descriptive or normative), (b) focus (e.g. denial), (c) context-specificity (e.g. flooding), (d) methodology and methods (e.g. qualitative and/or quantitative interviews), (e) time reference (e.g. denial in an early or later stage of a flooding) and (f) interpretations made (e.g. beneficial short-term denial vs. maladaptive long-term denial), we could map out our tensions and avenues for future research. For example, our following analytical framework explicitly included both descriptive and normative approaches to best capture and analyse the variety of mental barriers (e.g. context-dependent beneficial/maladaptive effects of interpretive denial vs. a priori maladaptive effects of literal denial) and attached greater importance to the barriers’ context and time reference. This example illustrates how (tacit) misunderstandings hampered the development of a shared analytical framework and how we finally resolved our differences.

At their first collaborative meeting (see Fig. 1), our three researchers apparently agree on the need to integrate the concept of cognition in SES research. However, when looking at their unspoken thoughts, it becomes obvious that they disagree significantly on the concept and underlying aspects. The researchers have different implicit assumptions about, for example, (a) where cognition is realised (in the brain, body and/or environment), (b) whether cognition includes or excludes emotion, and (c) whether cognition is or is not distinct from behaviour. They believe they are “speaking the same language” by using the same words for concepts (cognition, behaviour) and are conveying information properly, while, in fact, they are not. This misconception becomes evident during their follow-up meeting (see Fig. 1). The neuroscientist, for example, is confused about the philosopher’s suggestion to integrate emotions into studying cognition. The neuroscientist assumes that cognition and emotion are modular and independent from each other and that thinking can be explained without considering emotions. Likewise, the neuroscientist and the psychologist are puzzled by the philosopher’s approach to explicitly consider the biophysical environment. They do not know that the philosopher presumes that cognition also exists outside the human body. Conversely, the philosopher is confused by the psychologist’s statement to distinguish thinking from behaviour. We will revisit these diverging assumptions later when we discuss different paradigms of cognition and perspectives on behaviour and illustrate ways to communicate effectively.

### Why focus on communication?

As outlined by Bennett and Gadlin (2014, p. 359), it has become a cliché to attribute problems in collaborative research to “problems in communication”. We take this “cliché”-perception as support for the claims of the National Academy of Sciences (2005, p. 19) that effective communication “is at the heart of interdisciplinarity” and cuts across aspects such as team trust, shared visions and effective leadership. Further, Lyall et al. (2011, p. 59) elaborate that the difficulty of communication is “one of the key issues identified by many” in interdisciplinary research.

Theoretically speaking, the vital need for adequate verbal and written communication about cognition appears obvious. In order to prevent misunderstandings and communication breakdown, or to enable a quicker discovery and resolution of them, it is intellectually instructive to engage in mindful conversations. Inspired by the work of Nishishiba (2018), by “mindful” conversation we mean that communicators (a) are aware of communication pitfalls *and* (b) therefore clearly express assumptions, precisely explain what they mean by cognition (and other relevant terms) and ask others to do the same.

In practice, however, this task is far from trivial as it requires patience, self-awareness and time, but also intellectual humility (ability to recognise the limitations of own knowledge), intellectual flexibility (willingness to rethink own perspectives based on other people’s insights) and intellectual generosity (sincere acknowledgement of other people’s assumptions) (Klein 2014; see also O’Rourke et al. 2014).

We further argue that even with mindful communication skills, expertise in cognition and/or collaboration experience on other multifaceted concepts (e.g. resilience) do not necessarily mean that collaborators know *why* and *how* cognition and related terms entail communication challenges or *how* to overcome them. Rather, a nuanced view of the various meanings and assumptions associated with the concept of cognition is necessary to detect potential communication pitfalls, achieve the synergy of expertise from several specialisms and avoid achieving multidisciplinary juxtaposition rather than interdisciplinary integration.

The point of our paper is to make salient and raise awareness of both conflicting conceptions of cognition and related concepts. We further point out relevant potential communication pitfalls among researchers that must be tackled if collaboration is to succeed. Hence, our paper aims to be both conceptually informative and practically relevant to enable effective communication. We thus (a) inform about cognition being a container concept, (b) identify conceptual foundations that need explication, i.e. objects of investigation and levels of description, and (c) discuss the concept of rationality to illustrate means of overcoming communication failure based on the above conceptual insights.

We seek to encourage readers to reflect more deeply on the underestimated problem of poor communication and raise awareness of points of explication for putting cognition research into practice (e.g. developing efficient research agendas from the outset; see Box 1). We do not claim that effective communication solves the entire challenge of collaboration. Nor do we aspire to eliminate stimulating conceptual ambiguity. However, we do think that mindful communication lays the foundation for integrating soundly across different domains and, to follow Stokols et al. (2005, p. 212), that an appropriate balance should be found “between diversity and debate among investigators on the one hand, and intellectual integration and social support on the other”. Put simply, the problem is not an intellectual conflict (i.e. understanding cognition differently) but a *hidden* intellectual conflict (i.e. understanding cognition differently without realising and dealing with it).

This paper is of interest to researchers, scientists and practitioners who are or will be involved in inter- and transdisciplinary (SES) research projects involving the concept of cognition. Regardless of whether these collaborators are from the humanities or the natural, social and life sciences and have a profound background in the cognitive sciences, they are connected by their communicative engagement with cognition and willingness to collaborate. We do not argue against the benefits of disciplinary investigations in SES research, but this is not the concern of the present paper (for this see e.g. Østreng 2010). Also, we acknowledge that not only miscommunication but also a lack of important conceptual foundations (especially among collaborators with backgrounds in non-cognitive disciplines) can hamper research. The paper’s conceptual insights might also be informative in this regard and facilitate future work, e.g. in terms of helping teams raise important questions from the outset of their projects.

## Cognition in social**–**ecological research

Planet Earth has entered the ‘Anthropocene’ era—the age of mankind, in which humanity constitutes a significant force of change at the planetary scale (Crutzen 2002; Steffen et al. 2011). Addressing and understanding the roots of the Anthropocene—namely the processes leading to and involved in human behaviour—is one of the most important societal tasks for humankind, policymakers and the science community (Beratan 2007; Brondizio et al. 2016). To meet this challenge, scholars increasingly highlight the importance of taking human cognition into account. Cognitive processes are considered to play a crucial role in shaping human behaviour. Consequently, this means they are considered crucial for better explaining and understanding it. Milkoreit (2012), for example, argues that every human behaviour, whether individual or collective, is driven by a certain motivation. She also claims that everything humans do starts in the mind. Similarly, Lazarus (1999) illustrates that a person and the environment interact, but that it is the person who cognitively appraises what a certain situation signifies for personal well-being and how to respond to it. In a similar vein, Beratan (2007) points out that any and every decision process ultimately begins with a single human brain responding to some information. And, going in the same direction, von Kenemans and Ramsey (2013, p. XV) conclude that there is “no behavior without brain function and no variation in behavior without variation in brain function”.

There is broad scientific consensus that cognition and behaviour are (somehow) interrelated (Aizawa 2015). Some controversies remain, however, e.g. as to whether everything people do inevitably starts in the brain (which largely depends on the definition of cognition used, see “Explicate: Objects of investigation” section), whether cognition is distinct from behaviour, or if it is (a type of) behaviour (which largely depends on the definition of behaviour; see “Explicate: Levels of description” section). And yet, despite its important role in scientific research, the mind is one of the least understood parts of human beings and one of the most complex issues in today’s SES research on global challenges, like climate change, shrinking biodiversity, food insecurity and poverty (IPCC 2007; World Bank 2015). The following research questions illustrate this research gap: Why do people ignore climate change even though they assess it as a current, visible, local, personal threat and are deeply concerned (see e.g. Gifford 2011; Bercht 2017)? Why do people who survive catastrophic events become convinced that they are less likely to be affected by future ones (see e.g. Marshall 2014)? Why and how does poverty limit cognitive functions and directly affect cognitive control, intelligence and decision-making (see e.g. Mani et al. 2013)? Such research indicates that people are far from the idealised, perfect world where they think and act accurately and impartially. Challenges such as climate change are thus not only a social and ecological issue, but also—and this perspective has only recently gained attention—a cognitive one.

The cognitive sciences, including cognitive psychology, neuroscience, cognitive linguistics, artificial intelligence (AI) and philosophy, work to understand the mind, how it relates to and shapes human behaviour (Wilson and Keil 1999). These academic fields would thus appear to be the obvious places to find connections for integrating human cognition into SES research. However, SES researchers have only recently begun to address the role of cognitive processes more profoundly. Hukkinen (2014) attributes this lack of attention to the traditional and persistent SES research focus on the macro-level interplay between social and ecological systems. The rarity of micro-level considerations of the human mind at the individual level in SES research is not surprising, given the (philosophical) challenges of a mismatch in ontologies and epistemologies, the complex nature of cognitive processes, and the limitations in assessing and operationalising them. In recognising this micro-level gap, Hukkinen and others have started to clearly put cognition on their SES research agenda. For example, Hukkinen (2012, 2014) suggests combining the ontology (what an SES is) and epistemology (how knowledge is obtained about it) with approaches from the cognitive sciences (e.g. embodied cognition). Jones et al. (2016) review human values as cognitive drivers of change within SESs and how values themselves may change over time in response to system changes. Beratan (2007) integrates a cognitive lens in decision-making in SES, striving for a more practically useful approach to design for change while taking advantage of the human ability to deal with complexity and uncertainty. And Cundill et al. (2015) analyse how learning can enhance the resilience of ecosystem services.

In summary, we have to conclude that despite scholars are increasingly addressing cognition in SES research, a profound discussion of the potential pitfalls of miscommunication about cognition is still missing.

## Cognition as a container concept

**Fig. 2:**
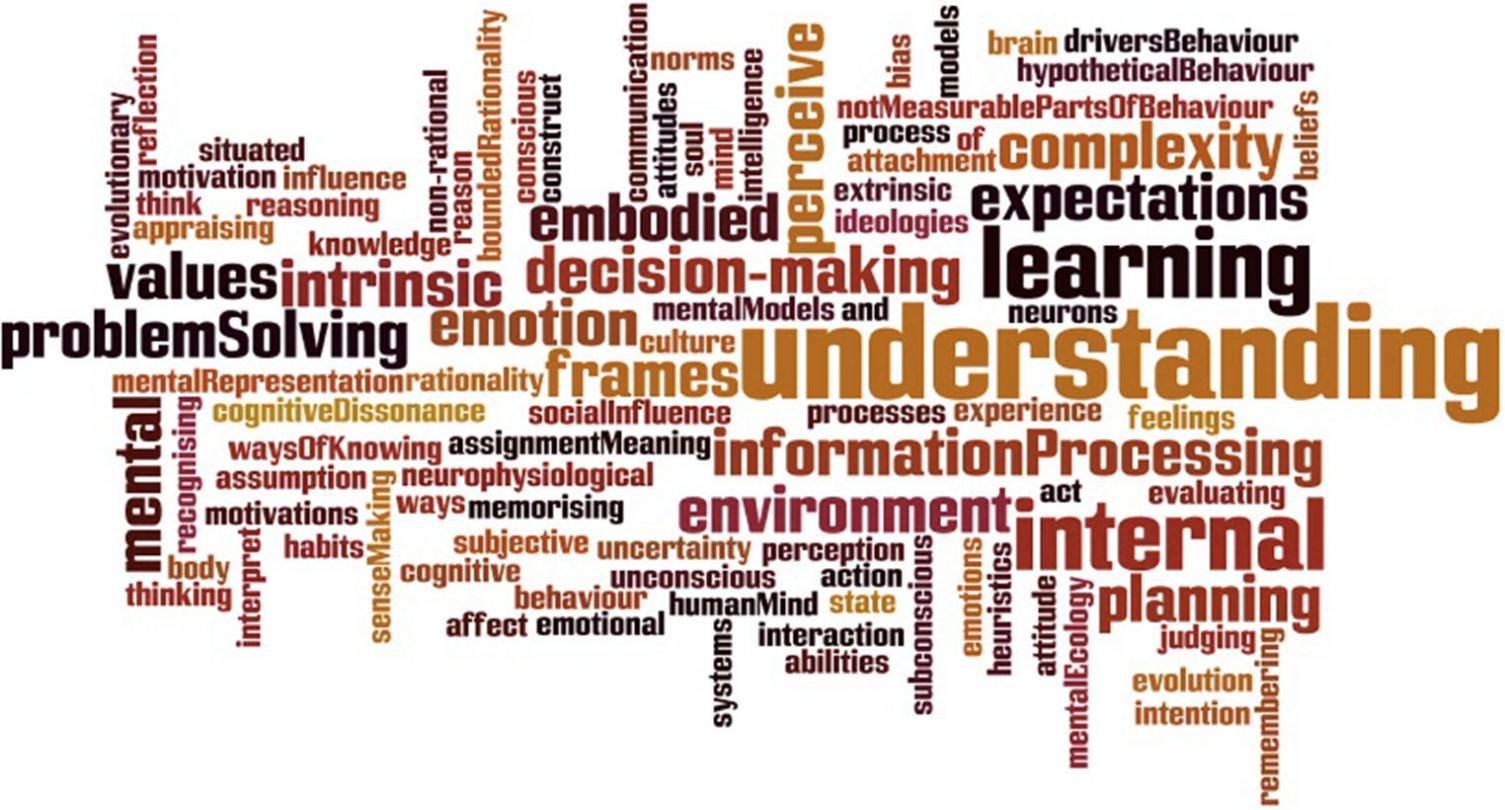
Brainstorming result on what SES researchers at Stockholm Resilience Centre associate with cognition

The notion of cognition is associated with many concepts. Figure 2 exemplifies this diversity of associations. They emerged from an informal brainstorming session among SES researchers in 2017 with different academic fields at the Stockholm Resilience Centre (SRC), Stockholm University. While the word cloud is not a precise measurement, it serves well to visualise and highlights the multidimensional nature of cognition.

There exist several particular definitions of cognition, but no general, universally accepted explanation or even a unified theory of cognition (Gershenson 2003). For instance, cognition has been defined as sensory information processing (LeDoux 1996), as the ability to solve a problem (Heylighen 1990), as the capacity to plan and structure behaviour for goal attainment (Toglia 2005) or as a “process of identifying, selecting, interpreting, storing, and using information to make sense of and interact with the physical and social world” (Kielhofner 2009, p. 85).

Etymologically, the term “cognition” comes from the Latin word *cognoscere*, meaning “to get to know”, and is generally used to mean something about what an entity knows or how it gets to that knowledge. It is, as Marcus (2009) summarises, the aim to figure out *what* the mind does and *how* the mind does whatever it does. From this perspective, and considering current literature (see Friedenberg and Silverman 2012 for an overview), cognition involves a collection of interacting, non-linear processes and components, like perception, attention, reasoning, learning, language, memory and decision-making, that gather, organise and convert information into knowledge and integrate it in existing experience. However, the definitions of all these component terms are rather vague and sometimes controversial themselves. While they provide an indication of what cognition is about, they also leave us asking what exactly concepts like “perception”, “attention” mean. Appendix S1 exemplifies how imprecise communication about different notions of perception can lead to frustrating misunderstandings.

In sum, cognition is a container concept that can refer to many different things, which makes it as powerful and weak as any other overarching concept. Arguing from a philosophical viewpoint, Gershenson (2003) points out that no entity *has* cognition as one of its intrinsic elements. Rather, cognition is *observed* and *described* inside a specific context to understand and explain a system. According to Gershenson (2003), cognition is therefore neither in the head nor in the world. It is instead an intangible construct simultaneously understood from many different schools of thought, which requires even more precise and clear communication. Not surprisingly, research on cognition thus not only differs in how cognition itself is defined but also in how research is performed. More precisely, differences exist in relation to the objects of investigation (where exactly do we observe and describe cognition?) and the levels of description used for explaining and predicting cognitive phenomena. Both are important aspects to be clear and precise about while researching and while communicating.

### Explicate: Objects of investigation

In relation to the different definitions of cognition, the focus on *what* is investigated varies tremendously. Questions, such as the following are relevant: What divides the non-cognitive from the cognitive? Where does human cognition stop and the rest of the world begin? Is cognition restricted to the boundary of the brain? If not, where else does it occur? And what role does cognition play in social interactions? Answers to these questions are not straightforward and highly debated. Yet, dealing with these issues is crucial for being able to refine and clarify the object of investigation in terms of research scope and focus (e.g. brain, body and/or environment) based on different paradigms and related conceptions of cognition.

To provide conceptual insight for avoiding possible communication pitfalls, Table 1 summarises the most common—partly overlapping and partly competing—paradigms in the contemporary cognitive sciences and their general underpinnings (including SES-oriented examples and key references). This summary is neither exhaustive nor final. Its sole purpose is to give an overview of the wide variety of approaches to cognition (Adams and Aizawa 2008). It thus provides a concrete way to make one’s position transparent to others, especially if one is not aware of other extant paradigms.

**Table 1:** Different paradigms for refining the object of investigation in cognition research

Paradigm	Conceptual viewpoint	Research scope and focus	Prominent literature examples and key further readings
Brain-bound cognition	Cognition only occurs within the brain	Cognitive processes in the brain	How cognition is brain-bound and why it does not extend from the nervous system into the body and environment (Rupert 2009); how the different processes occurring within brains share certain regularities that they do not share with extracranial systems such as lopping shears (Adams and Aizawa 2008); *Further reading:* Weiskopf (2008)
Embodied cognition	Cognition occurs within both the brain and the entire body of the organism	Interplay between cognition, the non-neural body and sensorimotor processes	How people remember more of the gist of a story when they physically act it out through improvisation (Scott et al. 2001; how people holding a *warm* cup of coffee view another person as more warm and caring than those holding a *cold* cup of coffee (Williams and Bargh 2008)); how eating salty pretzels, which activates the concept of thirst, leads to the belief that drought is a greater threat to humanity (Risen and Critcher 2011); how gesturing is not simply an aid to thinking but a part and parcel of the thinking (Clark 2008); *Further reading:* Glenberg (2010), Leitan and Chaffey (2014), Shapiro (2014)
Extended cognition	Cognition spatially extends beyond the boundary of the brain and body into the social/physical environment	Interplay between cognition, (the body) and the social/physical environment	How writing down a thought for future reference to be able not to retain it in the memory literally extends cognition into the environment such as a notebook (Clark and Chalmers 1998); how a “web-extended mind” or the idea that the technological and informational elements of the web serve as part of the mechanistic substrate that realizes human mental states and processes (Smart 2012); *Further reading:* Adams and Aizawa (2008), Clark (2008)
Emotional cognition	Cognition is not isolated from emotion	Interplay between cognition and emotion	How cognition and emotion jointly contribute to behaviour and why the neural basis of emotion and cognition should be viewed as non-modular (Pessoa 2008); how emotional and cognitive functions cannot be localized to specific brain areas (Kiverstein and Miller 2015); how decision-making is influenced by emotional factors (Thagard 2006); how emotional states selectively influence cognition-related neural activity in the lateral prefrontal cortex (Gray et al. 2002); *Further reading:* Moore and Oaksford (2002), Pessoa (2013)
Social cognition	Humans make sense of themselves and their social environment in order to coordinate with it	Role of cognition in social interactions	How people understand the minds and behaviour of themselves and others in order to interact with their social world (Fiske and Taylor 2016); how individuals mentally construct social reality (Greifeneder et al. 2004); how social comparison, mistrust and in-group beliefs impede climate action (Gifford et al. 2011; Marshall 2014, Kahan et al. 2011) *Further reading*: Carlston (2013), Devine et al. (1997)

Advocates of the classical cognitivist paradigm of brain-bound cognition maintain that cognition has to be understood as encapsulated by the organism’s central nervous system (Adams and Aizawa 2008; Rupert 2009). They acknowledge that cognitive processes within the brain causally interact with the body and the rest of the world (e.g. synaptic brain changes due to learning or social deprivation; visual and olfactory perception), but they argue that the body and world merely serve as sources of input and arenas for output. Put crudely, what is outside the brain is outside cognition.

In contrast, advocates of the emerging post-classical cognitivist paradigms support the idea that cognition spatially extends beyond the brain’s boundary. Criticising the mind–body dichotomy, e.g. the paradigm of embodied cognition claims that there is no sharp division between the brain, non-neural body and sensorimotor processes. From this perspective, cognition is embodied in the sense that the body “functions as a *constituent* of the mind rather than a perceiver and actor serving the mind, and is thus *directly* involved in, and productive of, cognition” (Leitan and Chaffey 2014, p. 3, emphasis by authors). Empirical evidence supporting this claim is provided, e.g. Williams and Bargh (2008) who discovered that people holding a warm cup of coffee view another person as more warm and caring than those holding a cold cup of coffee. Likewise, Risen and Critcher (2011) showed that eating salty pretzels, which activated the concept of thirst, led participants to believe that drought was a greater threat to humanity than other threats. These findings have important implications for how human beings perceive and appraise themselves in relation to the SESs in which they operate.

The paradigm of extended cognition goes yet a step further. While also incorporating the body and sensorimotor processes, it literally extends cognition beyond the organism’s skin into the organism’s environment (Clark 2008). To say cognition is extended means that it includes chunks of the environment as its partial realizer. Everyday examples would be reducing working memory load by taking notes during a conversation or consulting a notebook to insert and look up information (Clark and Chalmers 1998). According to this perspective, memory, information processing and knowledge are thus “outsourced” to the non-neural notes and notebook, waiting to be accessed when needed. As Smart (2012) points out in his work on the “web-extended mind”, such an approach heavily challenges “the notion that mind and cognition are solely internal (neural) phenomena by emphasizing the role played by extra-neural and extra-bodily factors in shaping the profile of much real-world cognitive processing.” (Smart 2012, p. 447f.). In a nutshell, extended cognition is treated as a joint product of brain, body and environment.

The paradigm of emotional cognition does not necessarily conflict with the previous paradigms (see e.g. Kiverstein and Miller 2015 on embodied cognition based on the inseparability of cognitive and emotional processing in the brain). It is generally less concerned about with exact “location” of cognition, but rather disagrees with treating cognition as independent from emotion. Whether one adopts a perspective that regards cognition and emotion as antagonists (e.g. LeDoux 1996) or instead as interactive or even inseparable (e.g. Pessoa 2008, 2013; Kiverstein and Miller 2015) largely depends on, firstly, the functional level of processing studied and, secondly, the definitions of both cognition and emotion adopted (Moors 2007). Gray et al. (2002, p. 4115), however, argue that “at some point of processing, functional specialization is lost, and emotion and cognition conjointly and equally contribute to the control of thought and behaviour”. Their statement summarises their findings concerning working memory performance influenced by mood. Moreover, similar research posits that emotion and cognition should be viewed as non-modular. There is, for example, evidence that decision-making (Thagard 2006) as well as climate change risk appraisals and policy support (Leiserowitz 2006) are significantly influenced by emotional factors.

Lastly, the paradigm of social cognition is also not necessarily exclusive from the paradigms above. It focuses particularly on the role of cognition in social interactions and sense-making. Accordingly, the way how people construct social reality and think about themselves and others plays a major role in how they think, feel and interact with the world around them (Fiske and Taylor 2016). Social cognition, as an area of study, embraces multiple aspects such as group belonging, social identity, social learning and cultural influence. Several studies (see Gifford et al. 2011; Kahan et al. 2011; Marshall 2014) have illustrated how, for example, social comparison (“Why should I act if *they* won’t act?”), mistrust (e.g. in climate science) and in-group beliefs (“We Republicans don’t worry about temperature increase.”) can impede climate action.

These paradigm examples indicate of how easily misunderstandings can occur, like in the conversation in the cartoon, Fig. 1. Mismatches are common, for example, between what a brain-bound cognitive scientist intends to express by saying “Cognition and SESs are interrelated” (cognition is brain-bound and interacts with but does not exist in the environment), and what a post-classical cognitive scientist believes to have been expressed by the same sentence (cognition is extended into and exists in the environment). The context of such communication pitfalls is essentially a social one. A question, like “Where is cognition located?”, may be interpreted differently, and may thus produce many valid answers, depending upon the extent to which one endorses the decomposability of the brain–body–environment system. Likewise, a comment like “In addition to cognition, a full account of SES research requires the consideration of emotions” might irritate an emotional cognition researcher who presumes that cognition and emotion belong together.

People—including scientists—use cognitive shortcuts when they are faced with multifaceted but familiar sounding concepts. They automatically clarify such terms for themselves in a way that corresponds with their assumptions and ingrained beliefs. These shortcuts, in turn, work as conceptual filters that distort information and knowledge exchange. Yet, if such conceptual filters are incongruent among communication partners and remain hidden, breakdown in communication is likely to occur. We therefore suggest to be explicit about one’s connection to a paradigm by using the relevant attributive adjective (e.g. brain-bound, embodied, emotional) to better qualify one’s definition of cognition (e.g. *brain*-*bound* cognition or *embodied* cognition instead of just cognition) and thereby improve effective communication.

### Explicate: Levels of description

The way scientific knowledge is generated is related to the level(s) of description a researcher uses. The study of the relationship between cognition and behaviour and the underlying mechanisms leading to behaviour can simultaneously employ different (hierarchical) levels of description or explanation at which scientists aim to describe and understand cognition and behaviour. As Newell (1990, p. 118) states, “[l]evels are clearly abstractions, being alternative ways of describing the same system, each level ignoring some of what is specified at the level beneath it”. There have been several proposals reflecting different numbers of levels of description (e.g. Pylyshyn 1984; Dennett 1987, 2009; Newell 1990). However, the main point is the widely agreed-upon notion that cognitive phenomena cannot solely be approached in neurological terms, i.e. cognitive science is moving away from positivist reduction of everything to basic physical or physiological laws). To enable explicitness for effective communication, we recommend communicating about which level(s) of description one’s research is described in. We follow the three levels of description of the philosopher and cognitive scientist Dennett (1987, 2009), as his approach is well known and has been widely used to describe (cognitive) systems and explain and/or predict (human) behaviour. He proposes three epistemologically independent stances: the physical, the design and the intentional level, detailed in Table 2.

**Table 2:** Levels of description for explaining and predicting behaviour based on Dennett (1987, 2009) and examples (provided by the authors)

Level of description	Level of abstraction	Domain	Concern	Examples
Physical stance	Most concrete (endpoint of ontological reduction within the study of cognition)	Physics, chemistry, biology, neurology	Explanation and prediction of behaviour based on chemical reactions, (neuro-) physiological properties (e.g. central nervous system, neurons, endocrine system) and physical laws (e.g. mass, energy, gravity); *understanding in terms of mechanisms*	We are taking the physical stance when we explain/predict: that an individual, with eyes open and facing a flood wave, will see that wave based on the interaction between physical energy, sensory coding and symbolic coding where a jumping individual is going to land based on his/her current trajectory how mountain ranges are formed based on plate tectonics when high tide will occur based on gravitational attraction
Design stance (functional)	Moderately abstract	Neuroscience, artificial intelligence, engineering	Explanation and prediction of behaviour solely based on knowledge or assumptions about the entity’s functional design and purpose; *understanding in terms of functions*	We are taking the design stance when we explain/predict: that an individual who saw a flood wave will be able to recall this event 1 day later on the basis of how the brain is “designed” to function (without any understanding of the physics and chemistry underlying recollection) that an air quality meter will start up and behave as designed when we press the “On” button that the lift will take us to the third floor when we push “3”
Intentional stance	Most abstract	Psychology, philosophy	Explanation and prediction of behaviour based on beliefs, desires and intentions that are ascribed to the entity in question (e.g. inferring from what we see and what we know of a situation and/or the person); *understanding in terms of agency*	We are taking the intentional stance when we explain/predict: that an individual will run away because (s)he knows a flood wave is coming and is afraid of getting caught by it that a fisher will head out to sea because (s)he wants to catch fish and believes that the storm is over that a chess-playing computer will win because it “wants” to win and “knows” how to make the wisest, most rational moves

Dennett (1987, 2009) argues that the more concrete the level of description, the more exact the predictions, at least in principle. Importantly, though, his understanding of behaviour is very broad. Thus, the internal process of perception or the formation of an inanimate object such as a mountain range would count as behaviour. We revisit this issue below after we presented examples of the different levels of description.

The most concrete and basic level of description is the *physical stance.* It makes explanations and predictions of behaviour based on information and knowledge of the chemical, physical and (neuro-)physiological properties of a given entity in conjunction with knowledge about physical laws. Accordingly, we can, for example, predict that an individual standing with his/her eyes open and facing a flood wave (sensory exposure) will actually see (i.e. physically and physiologically perceive) that wave based on the interaction between the brain’s sensory and perceptual system. The prediction of the individual’s behaviour (wave perception) is based on the knowledge that physical energy (sensory input) is transduced into sensory codes, which are then further transformed into highly abstract codes in terms of internal symbolic objects (i.e. the flood wave is the meaningful output; see also the tree-perception example from Appendix S1). In essence, the physical stance focuses on causal explanation informed by natural laws and determinism. As such, this stance is considered useful for explaining, for example, (neuro-)physiological malfunctions in a (cognitive) system or interpreting human behaviour as a result of mental illness (e.g. anxiety disorder) or physiological imbalance (see e.g. LeDoux 1996 on how adrenal and pituitary secretions that are elicited by long-existing stressful events can reduce learning and memory capabilities).

The next level of description is moderately more abstract and concerns the functional design of an entity. The *design stance* requires no insights into the entity’s (neuro-)physiological constitution or physical laws. Instead, explanations and predictions are solely based on knowledge or assumptions regarding the purpose of the entity’s “design”. Without any understanding of the neural networks underlying the long-term memory necessary, this allows for predicting, for example, that the individual seeing a flood wave will also be able to recall this event later because this is simply how the brain is “designed” to function. Likewise, when a fisher is out at sea without a computational navigation system, (s)he predicts that (s)he will be able to cognitively navigate her/his way back to land, not concerning herself/himself with the details of how spatial memories work in her/his hippocampus and medial temporal lobes and how they are summarised into a cognitive map. Importantly, however, predictions made from the design stance rest on at least two further assumptions: firstly, that the entity in question *is* designed as it is assumed to be; and secondly, that it will behave as it is “designed” *without* malfunctioning or any unforeseen disturbance (Dennett 2009).

The most abstract level of description is the *intentional stance* which requires no knowledge of physics, chemistry or design. Explanations and predictions are solely based on beliefs, intentions, goals and goal achievement capacities that are ascribed to the entity in question (e.g. inferring from what we see and what we know of a situation and/or the individual in a specific cultural/political/ecological context). As framed by Dennett (2009, p. 8), a “designed thing is treated as an agent of sorts, with beliefs and desires and enough rationality to do what it ought to do given those beliefs and desires”. We can, for example, predict that an individual will run away from a flood wave because we know that (s)he is seeing the wave coming and we know that (s)he most probably wants to avoid getting caught by that wave. The intentional stance has the advantage of great simplicity in terms of short-cutting, i.e. it is not concerned with the multitude of cause–effect relationships. However, zooming into the design or even physical level is considered helpful if predictions fail. For example, individuals who hardly engage with the topic of climate change even though they believe they should and also have the structural capacity to do so would be such a case in point. Gifford (2011) and Marshall (2014) reflect on a variety of common human mental barriers to engaging with climate change.

Dennett’s three levels of description exemplify one way of describing a (cognitive) system from different perspectives. One could argue that certain scientific disciplines match the different levels, e.g. psychology often uses the intentional level, neuroscience the design level and physics and biology the physiological/physical level (cf. also Table 2). However, we acknowledge that Dennett’s work is not without criticism (see e.g. Dahlbom 1993) and that there are other models of description (see literature references above). Yet, the point here is to demonstrate the general need to be as precise and clear as possible about the level of description one adopts.

A key point is that switching between levels can be problematic. For example, the perception of a flood wave (as defined above) is not predictable in terms of beliefs (intentional stance). Likewise, the fisher’s intention to go out to sea is not predictable in terms of (neuro-)physiological processes (physical stance) or the brain’s function (design stance). In addition, the design stance is riskier than the physical stance in terms of the extra assumptions made and the intentional stance is riskier than the design stance due to the assumption of free will (see Dennett 1987 for a more nuanced discussion). Also, each level differs in its underlying assumptions. Given this background, misunderstandings between communication partners who are either familiar or unfamiliar with Dennett’s levels of description are likely to occur if these communicators are not explicit about

- *the level of abstraction* they are referring to: Do they understand cognition and behaviour in terms of concrete sub-personal mechanisms (physical stance), somewhat concrete sub-personal functions (design stance) or abstract personal agency (intentional stance)?
- *the level of precision*, which their explanations/predictions are based on: How do they assess and evaluate (the risk of) incorrect predictions? Do they presume causality, regularity (physical stance) or rather correlation and more irregularity (design/intentional stance)?
- *the notion of behaviour* they are applying: Do they regard behaviour as overt (publicly observable; e.g. running away) and/or covert (only observable by the entity performing the behaviour; e.g. reasoning)? Or, put differently, do they understand physiological activities, cognitive processes or movements of inanimate objects/artefacts as a type of behaviour (e.g. cognitive behaviour) or as distinct from behaviour?

For example, epistemological confusion might arise if a neurologist thinks of explanation by causes (physical stance), while a psychologist thinks of explanation by reason (intentional stance). Determinists and libertarians often appear to be contradicting each other, but are, in fact, talking about different things. Yet, both perspectives may be equally valid, depending on the level of description. Likewise, confusion might arise if perception or reasoning are defined as behaviour on the one hand, and as non-behavioural processes on the other (see Fig. 1), or if predictions of behaviour are regarded either as rather safe or quite risky. People misunderstand each other if they do not know each other’s point of reference (e.g. safe physical stance versus risky intentional stance). Again, a solid foundation of concise communication and expressed assumptions allow for clarification and reduces the likelihood of misunderstandings.

### Detecting communication pitfalls of rationality

Falling under the umbrella of cognition as it is influenced by mental processes (Simon 1993), the widely used but ambiguous concept of rationality represents a prominent example of the need for mindful communication. Other examples could also have worked as illustrations (e.g. mental models, values, learning; see also Appendix S1 on misunderstandings and confusion about perception). However, we selected rationality for a more profound discussion because the rationality is often an underlying implicit assumption (Schlüter et al. 2017). In general, rationality reflects an evaluation of a behaviour and/or process leading to a behaviour in relation to its goals. However, closer scrutiny reveals that the concept’s meaning can differ greatly, depending on the different scientific assumptions that underlie the label of rationality and the different objects of evaluation.

#### Differences in scientific assumptions

Scientific assumptions regarding rationality distinguish between, for example, “logical” or “ecological” rationality (Gigerenzer and Gaissmaier 2011). Logical rationality reflects the more ‘traditional’ and often implied form of rationality. The concept is used to evaluate behaviour and its processes against the laws of logic and probability. Scholars use it to ask questions such as whether a behaviour is consistent, uses all information and corresponds to an optimum or ideal goal, i.e. the best solution available. In rational choice theory (RCT) and (neoclassical) economics, rationality assumes knowledge of all possible solutions and their consequences. Choosing is then merely a matter of maximising/optimising. Logical rationality is then determined as a derived evaluation that measures a deviation from the ideal or optimal. Ecological rationality, in contrast, evaluates behaviour and its process in relation to an agent’s environment(s) it performs in by either being good enough, i.e. satisficing, or being better than other behaviours/processes, i.e. competitive testing (Gigerenzer and Gaissmaier 2011). This approach assumes that individuals are boundedly rational and acknowledges time limitations (e.g. time available for making a decision), assets and capacities (e.g. limited knowledge and information processing that do not allow for finding an optimal solution) and the actual task environment (e.g. availability of solutions that might not even have an optimal solution; uncertainty) (Simon 1993; Gigerenzer and Brighton 2009).

We want to point out here that what is considered rational under the logical and normative account might not be considered rational under the ecological and contextual account, and vice versa. Often, however, the concept rationality is used without clearly communicating what kind of evaluation criteria one adopts. To illustrate, a given decision (e.g. fishing for salmon only) can be evaluated as either ecologically rational because it is better than other decisions (better, not best, because the optimal strategy is unknown to the decision-maker), or as logically irrational because it deviates from a predetermined norm or optimum (e.g. diversifying fish catch to buffer against market and ecosystem shifts). It is thus not the best imaginable decision. The same decision can consequently be evaluated both as rational and irrational depending on different underlying scientific assumptions. However, if these assumptions remain implicit in conversation, rationality easily becomes a confusing false friend (sounding alike, but having different meanings). As mentioned above, the application of attributive adjectives can increase preciseness and reduce miscommunication, i.e. using *ecological* rationality and *logical* rationality instead of just rationality. Even though researchers and practitioners might not know the concepts of logical and ecological rationality, they will detect from the added adjectives that each concept must have a different meaning. At best, they will be motivated to investigate more and enquire about the concept’s underlying assumptions for clarification.

#### Differences in the object of evaluation

Both behaviour and the processes leading to behaviour can be evaluated. On the one hand, substantive evaluation is used to evaluate whether the outcome of a given behaviour is appropriate to the achievement of given goals within the limits set by given conditions and constraints (Simon 1993). From this (economic) perspective, behaviour is substantively rational when it in fact achieves these goals. On the other hand, procedural evaluation is applied (e.g. in psychology) to evaluate the process (the “how”) that generated a behaviour. Put differently, the question is: What cognitive processes must take place so that goals/decisions will be reached? In this sense, as Simon (1979, p. 67) proposes, behaviour is described as irrational when it represents inappropriate reasoning or “impulsive response to affective mechanisms without an adequate intervention of thought”. This assumption, however, is also controversial; e.g. Kirman et al. (2010) hold the opinion that emotions do not necessarily interfere with rationality but rather can be central to it.

The crucial distinction—and common confusion—between these two kinds of evaluation rests on the distinct objects of evaluation (outcome or process), which is seldom addressed in discussions. As an example, based on Simon’s work (1993), decision-making and the rationality on which it is based involves a sequence of three (intertwined) steps. Firstly, finding and drawing attention to a problem (deciding which problem is the most important one is crucial in the decision-making process); secondly, thinking about what alternatives/kinds of solutions might deal with that problem; and thirdly, evaluating those alternatives/solutions and choosing among them. While the substantive evaluation concerns the latter (i.e. rationality of the decision itself), the procedural evaluation concerns all three (i.e. procedures used to reach the decision). Thus, depending on the object of evaluation, a behaviour that is procedurally rational (in terms of *how* the choice is taken, i.e. appropriate deliberation) is not necessarily substantively rational (because given goals are not achieved), and vice versa (e.g. achievement of given goals is based on inappropriate reasoning). For example, a coastal fisherman’s decision to head out to sea to catch fish can be both procedurally rational (he needs to catch fish because he lives off fishing and has no other alternative income sources) and substantively irrational (he will not catch much fish due to overfishing). Explicitness about the object of evaluation one is referring to is essential to avoid misunderstandings (e.g. about whether the fisherman’s behaviour is rational or irrational). This can be achieved, for example, by applying attributive adjectives such as *procedural* rationality and *substantive* rationality.

## Conclusion and outlook

This paper argues that, often, researchers and practitioners seem not to realise that communication about cognition is failing more often and more easily than they imagine. Even though they are aware of the concept’s multitude of definitions, they are and cannot be aware of *all* its different facets and related communication pitfalls. With any word or phrase such as cognition, perception or rationality that appears familiar, a listener automatically attributes meaning to it, often without this attribution process being conscious or expressed. Yet, this lack of communicative clarity becomes problematic if, firstly, this meaning differs from the meaning the speaker intended and if, secondly, such a meaning discrepancy remains unresolved or is not taken into account sufficiently. In those cases, multifaceted words or phrases easily become false friends (sounding similar, but with different meanings or scope of usage; e.g. logical versus ecological rationality) and misunderstandings arise around the object of investigation (e.g. is cognition constituted only in the brain or also beyond it?) and the level of description at which cognition and behaviour are analysed (e.g. Dennett’s physical, design and intentional stances). This can slow down collaboration and progress and become a source of conflict and frustration.

To be clear, we do not advocate a common, universally accepted or final definition of cognition and its related concepts. Nor do we argue for a common understanding of which theories or conceptual frameworks are best suited to approach cognition. These may well be impossible goals and would also probably reflect arbitrary one-sidedness and hamper research-enhancing critical debate. Rather, we suggest that the challenges of addressing cognition in SES research are not met by universal definitions and understandings, but by clear, precise, explicit and direct communication of all relevant conceptual, ontological and epistemological assumptions, including knowledge gaps and uncertainties, which accompany this integrative journey.

In order to prevent misunderstandings or more quickly discover and resolve them, it is not only important to be aware of, and explicit about, one’s own assumptions and presumptions, but also to familiarise with those of the communication partners and to listen/read carefully and actively. This entails making a conscious effort not only to hear or read the words that another person is saying or writing but also to appreciate the complete message being sent and, if necessary, to ask clarification questions (e.g. “Is this what you mean by ‘rationality’?”). It is thus essential to “mind”, i.e. to recognise what others think, and vice versa to reflect upon where oneself stands in relation to other assumptions and viewpoints. As explicated by the Theory of Mind (ToM), proposed by Premack and Woodruff (1978), humans generally have the innate cognitive ability to attribute mental states such as thoughts, beliefs, assumptions, intentions and emotions to oneself and to others. This is what makes us social beings. However, successful interaction with others through the Theory of Mind crucially depends on proper communication, especially for fuzzy topics such as cognition.

Our paper aims to encourage researchers and practitioners to make full use of their ability to be mindful and to ensure (greater) communicative clarity, particularly in terms of the overall ontological assumptions of what cognition is and the epistemological assumptions of how to obtain knowledge about cognition. In sum, mindful and unambiguous verbal and written communication and a frequent revisit of relevant own and other viewpoints are especially needed with regard to

- the notion of cognition itself (e.g. is cognition considered either as distinct from behaviour or as a type of behaviour);
- other concepts used to further specify cognition (e.g. perception and whether perception is seen as stimulus-, representation- or evaluation-driven; see Appendix S1);
- the paradigms applied to refine the object of investigation (e.g. brain-bound, embodied, extended, emotional and/or social cognition);
- the levels of description used to describe and study cognition and behaviour (e.g. by explicitly referring to Dennett’s levels of description and three stances);
- all other concepts used that fall under the umbrella of cognition (e.g. logical versus ecological rationality; substantive versus procedural evaluation of rationality).

In conclusion, we hope this paper will serve as a foundation for more constructive and conceptually oriented dialogue among SES researchers and practitioners, and enable more fruitful collaboration. Mindful communication which is best characterised by openness and tolerance towards different, but equally valid assumptions regarding cognition, represents a crucial milestone on the way to successfully integrating cognition in SES research. Overall, we advocate to mind the “mind” in two intertwined ways: firstly, consider the important role of cognition in current SES research and, secondly, be aware of the mind in terms of misinterpretations and misunderstandings caused by unmindful communication.

## Electronic supplementary material

Below is the link to the electronic supplementary material.

Supplementary material 1 (PDF 9 kb)
