# Supplementary material for: Mind the mind: How to effectively communicate about cognition in social–ecological systems research
Source: Ambio. 2018 Sep 22;48(6):590–604. doi: 10.1007/s13280-018-1099-7 (PMC6486894; doi:10.1007/s13280-018-1099-7)
Supplement: Supplementary file 1 — Supplementary material 1 (PDF 9 kb) [file 13280_2018_1099_MOESM1_ESM.pdf]

Ambio

Electronic Supplementary Material
This supplementary material has not been peer reviewed.

Title: **Mind the mind: How to effectively communicate about cognition in social-ecological systems research**

Authors: Anna Lena Bercht, Nanda Wijermans

Appendix S1

The following example of the perception of nature illustrates how imprecise communication about different notions of perception can lead to frustrating misunderstandings. From a "common-sense" point of view (naïve realism), perception is stimulus-driven and mirrors the external, mind-independent world as it really is (i.e. perception of input, e.g. the image of a tree). From a perceptual psychology point of view, however, perception is representation-driven rather than stimulus-driven and mirrors the way perceptual representations as an entirety are organized in the mind as meaningful categories (Mausfeld 2010). More precisely, the input is not a tree, but a specific physical spatio-temporal energy pattern on the retina. It is then the entire perceptual architecture of the brain including its interfaces with the sensory system, the motor system and the higher cognitive system (neocortex) that imposes a structure on the sensory input. This structure cannot be derived from an analysis of the physical input alone (i.e. perception of output not input; a tree is the meaningful product of perception, not the antecedent). From a human geography perspective, perception is considered equivalent to evaluation. In this sense, the question "How do people perceive trees?" actually asks how people appraise trees, for example in terms of personal significance for their well-being (note e.g. the idea of risk perception which actually refers to appraisal of risk as a mental construct).

Evidently, the same term (perception) can have different meanings to different people in different contexts. Depending on distinct, often implicit, assumptions, a tree can be seen either as the input (e.g. by an ecologist), outcome (e.g. by a psychologist), or evaluative object of perception (e.g. by a human geographer). If these different scientists do not clearly communicate the different meanings they have in mind, misunderstandings are inevitable to occur (e.g. on whether a research focus should be more on nature as input and less on people's subjective sense-making, and vice versa). In the perception example, clear communication of one's viewpoints is especially needed with regard to conceptual operationalisation (e.g.

perception as stimulus-, representation- or evaluation-driven?), relevant ontological considerations (e.g. does perception correspond to an objective world?), and epistemological considerations (e.g. how can perception give us knowledge and justified beliefs about things outside of ourselves?).

References

Mausfeld, R. 2010. The perception of material qualities and the internal semantics of the perceptual system. In *Perception beyond inference. The information content of visual processes,* ed. L. Albertazzi, G. van Tonder, and D. Vishwanath, 159-200. Cambridge: MIT Press.
